# *In silico* gene expression profiling in *Cannabis sativa*

**DOI:** 10.12688/f1000research.10631.1

**Published:** 2017-01-23

**Authors:** Luca Massimino

**Affiliations:** 1 Molecular Oncology Unit, San Gerardo Hospital, Monza, Italy

**Keywords:** Cannabis sativa, gene expression, cannabinoid pathway

## Abstract

The cannabis plant and its active ingredients (i.e., cannabinoids and terpenoids) have been socially stigmatized for half a century. Luckily, with more than 430,000 published scientific papers and about 600 ongoing and completed clinical trials, nowadays cannabis is employed for the treatment of many different medical conditions. Nevertheless, even if a large amount of high-throughput functional genomic data exists, most researchers feature a strong background in molecular biology but lack advanced bioinformatics skills. In this work, publicly available gene expression datasets have been analyzed giving rise to a total of 40,224 gene expression profiles taken from cannabis plant tissue at different developmental stages. The resource presented here will provide researchers with a starting point for future investigations with *Cannabis sativa*.

## Introduction

The cannabis plant has been used for medical purposes for centuries, before being socially stigmatized for the last half century ^1^. Nevertheless, more than 430,000 published scientific papers exist, with about 25,600 works published in 2016 ( https://scholar.google.com/). In addition, there are about 600 ongoing and completed clinical trials involving cannabis ( https://www.clinicaltrials.gov/).

The endocannabinoid system is involved in virtually every biological function ^2^, so it is not surprising that cannabis is being used to treat neurological ^3^, psychiatric ^4^, immunological ^5^, cardiovascular ^6^, gastrointestinal ^7^, and oncological ^8^ conditions.

Today, a large amount of high-throughput functional genomic data exists. Nonetheless, even in the era of ‘omics, the great majority of researchers feature a strong background in molecular biology but lack advanced bioinformatics skills ^9^.

In the present work, publicly available gene expression data taken from cannabis plant tissue at different developmental stages (shoot, root, stem, young and mature leaf, early-, mid- and mature-stage flower) have been analyzed, giving rise to 40,224 gene expression profiles. Moreover, the expression patterns of 23 cannabinoid pathway related genes are described. The data note provided here will aid future studies by providing researchers with a powerful resource for future investigations.

## Material and methods

### Gene expression analysis

Gene expression datasets were downloaded from the NCBI SRA directory ^10^ ( https://www.ncbi.nlm.nih.gov/sra/) with accession numbers SRP006678 and SRP008673. Raw sequences were mapped to the canSat3 reference genome ^11^ with TopHat2 v2.1.0 ^12^. Gene counts and relative transcript levels were obtained with Cufflinks v2.2.1.0 ^13^, and submitted to NCBI GEO ( https://www.ncbi.nlm.nih.gov/geo/) with accession number GSE93201. Cannabinoid related genes were found within the canSat3 transcripts with the Cannabis genome browser BLAT web tool ^11^ ( http://genome.ccbr.utoronto.ca/cgi-bin/hgBlat?command=start). Gene expression heatmaps and unsupervised hierarchical clustering were carried out with GENE-E ^14^.

## Results

**Figure 1.**
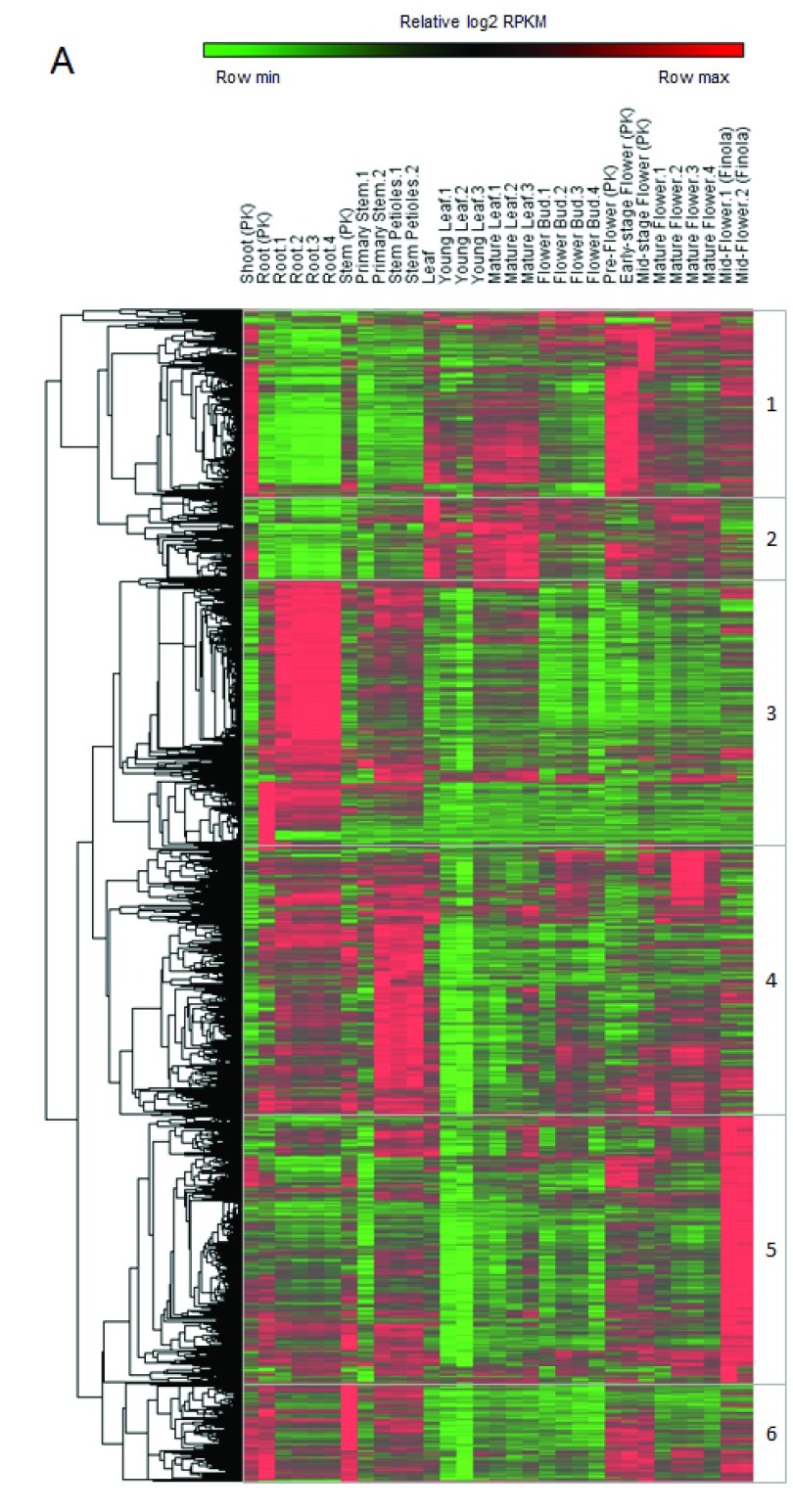
Gene expression profiles taken from cannabis plant tissue at different developmental stages. Heatmap showing relative expression values (log2 RPKM) of the highest expressed genes. Six gene clusters were defined in accordance with the unsupervised hierarchical clustering.

The *Cannabis sativa* reference genome and transcriptome have been published, although data analysis is still at the preliminary stages ^11^. In other words, we know what the presumptive genes are, but we do not know the chromosomes they are located in, nor their molecular functions. Given that this high-throughput gene expression data is publicly available, expression analysis of these yet unidentified genes can be performed. To this end, public repositories have been surveyed for transcriptional profiling datasets derived from *Cannabis sativa*. In total, 31 RNA-seq datasets derived from one hemp and two different psychoactive strains (NCBI SRA accession numbers: SRP006678 and SRP008673) of *Cannabis sativa* shoot, root, stem, young and mature leaf, early-, mid- and mature-stage flower have been analyzed. Unsupervised hierarchical clustering of gene expression values revealed six clusters of genes with specific tissue/stage expression ( Figure 1). Cluster 1 genes display high expression levels in shoots, mature leaves, and flowers; cluster 2 genes in leaves and flowers; cluster 3 genes in roots and stems; cluster 4 genes in roots, stems, and flowers; cluster 5 genes in hemp flowers and cluster 6 genes in shoots, roots, stems, and flowers.

**Figure 2.**
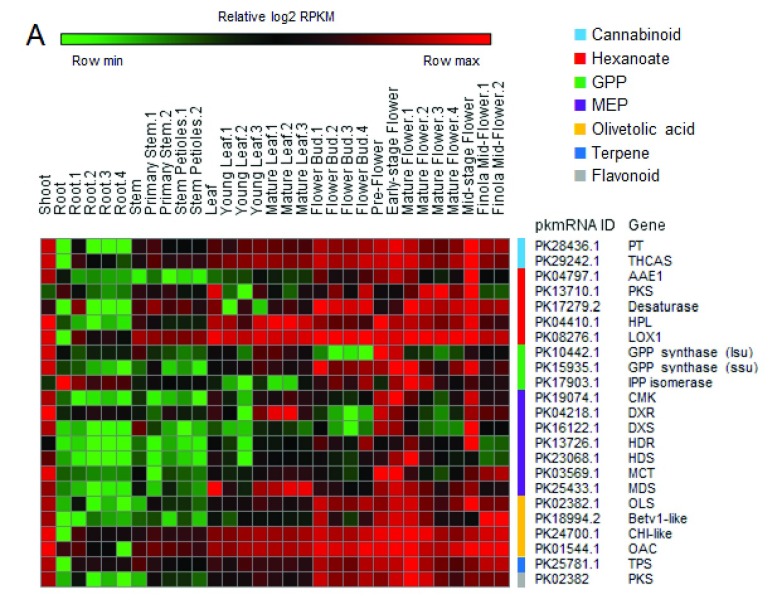
Gene expression analysis of the cannabinoid pathway. Heatmap showing relative expression values (log2 RPKM) of genes belonging to cannabinoid and precursor (hexanoate, GPP, MEP, olivetolic acid) pathways, together with terpene synthase (TPS) and polyketide synthase (PKS).

Genes involved in the biosynthesis of cannabinoids and their precursors have been shown to be overexpressed in flowers ^15^. To validate gene expression profiling, cannabinoid, hexanoate, 2-C-methyl-D-erythritol 4-phosphate ( *MEP*) and geranyl diphosphate ( *GPP)* pathway genes ^11, 16^, together with the olivetol synthase ( *OL*S) gene ^17, 18^, the (-)-limonene terpene synthase ( *TPS*) gene ^19^ and the polyketide synthase ( *PKS*) gene ^20^, have been analyzed. As expected, most of these genes were overexpressed in flowers, although many of the genes also displayed high expression in other tissues ( Figure 2; Supplementary table 1). Interestingly, virtually all of them were highly expressed in the shoot.

## Discussion

Today, cannabis and its derivatives are successfully employed for treatment of a large number of different pathological conditions ^3, 5– 8^. Each year, more articles related to cannabis are published, with about 25,600 studies published in 2016 ( https://scholar.google.com/). Remarkably, only 3% of these papers (13,300 out of 432,000) also take genomics into consideration, with very few of them directly relating to the genomics of cannabis. This could be due to the fact that, for obvious reasons, most researchers still lack advanced bioinformatics skills and are therefore limited in their research ^9^.

To this end, a total of 40,224 gene expression profiles taken from cannabis plant tissue at different developmental stages were obtained by exploiting common bioinformatics pipelines ^13^. Moreover, expression profiles of the genes belonging to the cannabinoid pathway ^11, 16– 20^ are provided.

Even if these data are preliminary, some observations can already be made. For instance, virtually all genes found to be highly expressed in flowers ( Figure 1, cluster 1 and Figure 2) also displayed high expression in the shoot. Having had only one sample at this specific developmental stage, these results could be derived from technical issues rather than differences in gene expression. However, not all transcripts (57%) were found to be overexpressed in the shoot, thus pointing toward the possible specificity of these changes. If this is confirmed, it may provide researchers with the possibility to study the molecular function of flower specific genes directly in sprouting plants, without having to wait for the plant to fully bloom.

*Cannabis sativa* is a versatile plant - it is being used for medical as well as for industrial purposes ^21, 22^. For this reason, cutting-edge genomics technology is currently being applied either to ameliorate specific phenotypes, or for breeding purposes ^22– 27^. Cluster 5 genes ( Figure 1) seem of great interest in this regard, as they are visibly overexpressed specifically in non-psychoactive cannabis flowers. These genes could be downregulated in hemp in order to create new strains high in cannabidiol (CBD), but with the proper *entourage* effect commonly found in the psychoactive counterparts ^28^. On the other hand, hemp specific genes could be upregulated in marijuana to produce high fiber/oil containing crops harboring therapeutically valuable active principles within their flowers. One potential candidate is the *Csfad2a* gene which was recently found to be highly expressed only in some hemp strains. Here, high *Csfad2a* expression was correlated with both higher oil content and lower oxidation tendency, eventually leading to the production of a significantly better commercial product ^26^.

Perhaps the major pitfall of this kind of analysis comes from the fact that although the current cannabis reference genome and transcriptome have been published, data analysis is still at the preliminary stages ^11^. Like in other plants, the cannabis genome is highly redundant and difficult to resolve ^29^. It is very likely that false negatives have caused important transcripts to still be missing. Nevertheless, these 40,224 gene expression profiles will provide researchers with a valuable resource and important genomic insights for future investigations with *Cannabis sativa*.

## Data availability

The data referenced by this article are under copyright with the following copyright statement: Copyright: © 2017 Massimino L

Raw expression data can be found in the NCBI SRA directory ( https://www.ncbi.nlm.nih.gov/sra/) with accession numbers SRP006678 and SRP008673.

Processed data can be found in the NCBI GEO repository ( https://www.ncbi.nlm.nih.gov/geo/) with accession number GSE93201.

## Supplementary material

**Supplementary table 1. Cannabinoid metabolism related gene profiling in different tissues and developmental stages.** Gene expression matrix of cannabinoid pathway genes. Expression values are expressed in RPKM.
