# Supplementary material for: In silico gene expression profiling in Cannabis sativa
Source: F1000Res. 2017 Jan 23;6:69. [Version 1] doi: 10.12688/f1000research.10631.1 (PMC5414814; doi:10.12688/f1000research.10631.1)
Supplement: Peer review history

# Peer review: Referee response for version 1

**DOI:** 10.5256/f1000research.11455.r20301

**Published:** 2017-05-02

**Authors:** Sergio Esposito

**Affiliations:** 1 Dipartimento di Biologia, University of Naples Federico II, Napoli, Italy

The bioinformatic approach presented in this study is potentially highly interesting, providing to scientists a useful data set to investigate the different pathways activated in *Cannabis sativa* at different developmental stages, and in different organs.

 I would only ask to the Author if some of the gene sets identified could be further divided into sub-clusters: e.g. there is a “green” area in Fig.1 – cluster 4 in leaves and flower buds (about in the second quarter from above). On the opposite, this area is "red" in cluster 5 – first quarter. Could be these results interpreted under the light of the considerations exposed in the discussion?

I have read this submission. I believe that I have an appropriate level of expertise to confirm that it is of an acceptable scientific standard.

# Peer review: Referee response for version 1

**DOI:** 10.5256/f1000research.11455.r20299

**Published:** 2017-02-20

**Authors:** Gea Guerriero

**Affiliations:** 1 Environmental Research and Innovation (ERIN), Luxembourg Institute of Science and Technology (LIST), L-4362 Esch/Alzette, Luxembourg

The present study suits the Data Note format and can be a useful resource for future studies centered on *Cannabis sativa.* I find the bioinformatics approach sound.  I have one suggestion for the author. How about enriching Figure 1 with a representation of GO/pathway enrichment analysis for each cluster (for example with ClueGO in Cytoscape; Bindea et al., 2009 ^1^)?

 The interest around this multi-purpose crop is increasing and recently transcriptomics data have been published for a fiber variety too (see for example Behr et al., 2016 ^2^). The approach described in this note can be applied also to other varieties in the future and/or to other tissue types.

I have read this submission. I believe that I have an appropriate level of expertise to confirm that it is of an acceptable scientific standard.

## References

[1] BindeaGMlecnikBHacklHCharoentongPTosoliniMKirilovskyAFridmanWHPagèsFTrajanoskiZGalonJ: ClueGO: a Cytoscape plug-in to decipher functionally grouped gene ontology and pathway annotation networks. *Bioinformatics*.2009;25(8) : 10.1093/bioinformatics/btp101 1091-3 10.1093/bioinformatics/btp101 19237447PMC2666812

[2] BehrMLegaySŽižkováEMotykaVDobrevPIHausmanJFLuttsSGuerrieroG: Studying Secondary Growth and Bast Fiber Development: The Hemp Hypocotyl Peeks behind the Wall. *Front Plant Sci*.2016;7: 10.3389/fpls.2016.01733 1733 10.3389/fpls.2016.01733 27917184PMC5114303
